# Contrasting patterns of density‐dependent selection at different life stages can create more than one fast–slow axis of life‐history variation

**DOI:** 10.1002/ece3.6122

**Published:** 2020-03-01

**Authors:** Jonathan Wright, Erik Blystad Solbu, Steinar Engen

**Affiliations:** ^1^ Department of Biology Centre for Biodiversity Dynamics Norwegian University of Science and Technology (NTNU) Trondheim Norway; ^2^ Department of Landscape and Biodiversity Norwegian Institute of Bioeconomy Research (NIBIO) Trondheim Norway; ^3^ Department of Mathematics Centre for Biodiversity Dynamics Norwegian University of Science and Technology (NTNU) Trondheim Norway

**Keywords:** carrying capacity, eco‐evolutionary dynamics, environmental stochasticity, life‐history evolution, population regulation, reproduction selection, survival selection

## Abstract

There has been much recent research interest in the existence of a major axis of life‐history variation along a fast–slow continuum within almost all major taxonomic groups. Eco‐evolutionary models of density‐dependent selection provide a general explanation for such observations of interspecific variation in the "pace of life." One issue, however, is that some large‐bodied long‐lived “slow” species (e.g., trees and large fish) often show an explosive “fast” type of reproduction with many small offspring, and species with “fast” adult life stages can have comparatively “slow” offspring life stages (e.g., mayflies). We attempt to explain such life‐history evolution using the same eco‐evolutionary modeling approach but with two life stages, separating adult reproductive strategies from offspring survival strategies. When the population dynamics in the two life stages are closely linked and affect each other, density‐dependent selection occurs in parallel on both reproduction and survival, producing the usual one‐dimensional fast–slow continuum (e.g., houseflies to blue whales). However, strong density dependence at either the adult reproduction or offspring survival life stage creates quasi‐independent population dynamics, allowing fast‐type reproduction alongside slow‐type survival (e.g., trees and large fish), or the perhaps rarer slow‐type reproduction alongside fast‐type survival (e.g., mayflies—short‐lived adults producing few long‐lived offspring). Therefore, most types of species life histories in nature can potentially be explained via the eco‐evolutionary consequences of density‐dependent selection given the possible separation of demographic effects at different life stages.

## Graphical abstract

Our model demonstrates how density dependence can uncouple the eco‐evolutionary dynamics of adult reproduction versus offspring survival to allow independent life‐history (fast vs. slow) evolution at different life stages. The normal simple fast–slow life‐history continuum can thus be modified into more complex life histories, such as those seen in most trees and large fish with large‐bodied long‐lived “slow” adults but explosive “fast” reproduction with many small offspring. We therefore show that fast–slow density‐dependent selection theory has the potential to explain even the most complex types of life‐history strategies seen in nature.

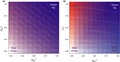

## 1 INTRODUCTION

Life‐history traits, such as reproductive rate and lifespan, are the product of inherently complex eco‐evolutionary dynamics (Hendry, 2016; Sæther, Visser, Grøten, & Engen, 2016). This is because the effects of natural selection on such traits will depend upon local population densities, and then, the values of such traits will feed directly back into the ecological dynamics of the populations within which they evolve. Processes such as density‐dependent selection and their consequences therefore need to be properly understood, especially if we are to accurately predict how natural populations of the same and of different species will respond to environmental change (Moritz & Agudo, 2013).

The challenge in understanding life‐history evolution as a result of density‐dependent selection received an early boost with the notion of *r*‐ versus *K*‐selection by MacArthur (1962), and more completely by MacArthur and Wilson (1967). High intrinsic (density‐independent) rates of reproduction should be favored in new and/or small populations, whereas in large established populations there should be density‐dependent selection to minimize any detrimental effects on fitness of intraspecific competition when populations approach carrying capacity. It was then suggested by Pianka (1970) and Roughgarden (1971) that species or populations experiencing density‐independent selection should therefore evolve faster life histories with limited parental investment per offspring and short adult life spans, while density‐dependent selection should promote greater levels of somatic and parental investment and slower life histories, with a number of early formal mathematical treatments (reviewed by Joshi, Prasad, & Shakarad, 2001).

Unfortunately, *r*‐ versus *K*‐selection theory was not explicit enough concerning the demographic mechanisms involved in any density‐independent and density‐dependent selection, and so it was difficult to confirm empirically or to predict exactly which life‐history traits should be implicated (Charlesworth, 1980; Stearns, 1993). As a result, work on density‐dependent selection of life histories declined, and this was exacerbated by the mathematical complexity of the eco‐evolutionary dynamics inherent in such issues (see Boyce, 1984; Reznick, Bryant, & Bashey, 2002). Our understanding of density‐dependent selection has since been helped by evolutionary models of specific life‐history traits like senescence (Abrams, 1993), which have even found some empirical support (e.g., Reznick, Bryant, Roff, Ghalambor, & Ghalambor, 2004). The rise of adaptive dynamics modeling has also provided some useful insights into eco‐evolutionary dynamics of life‐history trait evolution, especially with regard to the separation of density‐independent versus density‐dependent effects (Marty, Dieckmann, Rochet, & Ernande, 2011). However, a more general solution has recently been offered by mathematical developments of density‐dependent selection theory, which incorporate environmentally induced stochastic reductions in population size into models of life‐history eco‐evolutionary dynamics (Engen, Lande, & Sæther, 2013; Lande, Engen, & Sæther, 2009).

In such models, populations kept small by environmental stochasticity produce conditions that favor density‐independent selected life histories, because genotypes with high rates of reproduction at low population densities (*r*_0_) will contribute disproportionately to any population growth. Conversely, populations experiencing low levels of environmental stochasticity ultimately approach carrying capacity, where density‐dependent selected life histories are favored because of their ability to mitigate the density‐dependent effects that decrease fitness (*γ*). This allows such density‐dependent selected genotypes to contribute more offspring to the next generation in dense populations (Engen et al., 2013; Lande et al., 2009). A recent study on great tits (*Parus major*) has confirmed these model predictions in that females laying the largest clutch sizes at small population sizes were also the ones that experienced the greatest density‐dependent reductions in fitness (Sæther et al., 2016). Understanding the eco‐evolutionary dynamics of life‐history evolution therefore requires an appreciation of the degree to which population densities are limited by environmental (or demographic) stochasticity versus the limiting effects of density dependence. The predictions concerning precisely which life‐history (and other physiological and behavioral) traits we expect to be implicated in any particular system thus depend upon the contributions of each trait to variation in density‐independent reproduction (*r*_0_) and density‐dependent reductions in fitness (*γ*)—see Wright, Araya‐Aroy, Bolstad, and Dingemanse (2019). This is because selection will maximize Malthusian fitness given the trade‐off between these two parameters, which is what defines this revised and fully eco‐evolutionary version of density‐independent versus density‐dependent selection (Engen & Sæther, 2017).

Importantly, these recent advancements in density‐dependent selection theory provide a general and conceptually robust explanation for the fast–slow continuum observed in life‐history variation. Despite Pianka's (1970) overly simplistic original dichotomy or gradient between *r*‐selected versus *K*‐selected species, a “pace‐of‐life” fast–slow continuum has now been identified as a major axis of phenotypic variation in key life‐history traits in birds (Sæther, 1987; Sæther & Bakke, 2000), mammals (Bielby et al., 2007; Gaillard et al., 2005; Oli, 2004; Stearns, 1983), fish (Bjørkvoll et al., 2012; Goodwin, Grant, Perry, Dulvy, & Reynolds, 2006), and reptiles (Bauwens & Diaz‐Uriarte, 1997), and more recently across all animals (Healy, Ezard, Jones, Salguero‐Gómez, & Buckley, 2019) and in plants (Adler et al., 2014; Salguero‐Gómez et al., 2015). However, rather like the initial success of *r*‐ versus *K*‐selected species that then failed to explain life‐history variation in as many as 50% of species (Stearns, 1977, 1993; Wilbur, Tinkle, & Collins, 1974), this more recent fast–slow continuum likely also fails to account for much of the natural variation we see in life histories. For example, some long‐lived species (e.g., trees and large fish) appear density‐dependent selected, but show explosive density‐*in*dependent selected reproduction involving many small offspring (see Winemiller, 2005). This has led to statistical attempts to identify multiple dimensions in life‐history variation, such as a “first order tactic” in allometry variation (i.e., autosomal investment in body size), a “second order tactic” of variation in the timescale of demographic turnover (i.e., generation time), and a “third order tactic” in the degree of iteroparity (see Gaillard et al., 1989). However, these efforts still reflect different aspects of what is essentially a unidimensional fast–slow axis of life‐history variation. We currently lack a theoretical framework that allows us to understand and predict the full range of life‐history strategies in nature and especially the examples of density‐independent versus density‐dependent selection apparently working in opposite directions at different life stages within the same life history.

It was for exactly these reasons that life‐history theorists abandoned *r*‐ versus *K*‐selection and turned to age‐structured demographic models. This made it possible to explore a wider range of life‐history variation by considering different life stages (see Reznick et al., 2002). The problem was that early demographic models did not include density dependence, and those later ones that did could not properly explore the eco‐evolutionary consequences of any age structure on the overall evolution of the life history. Reznick et al. (2002) provides an informal attempt to reconcile *r*‐ versus *K*‐selection theory with the demographic models that replaced it in order to explain life‐history trait variation according to contrasting levels of density‐dependent selection in different populations of guppies (*Poecilia reticulata*). A formal solution here would be to combine recent eco‐evolutionary models of density‐dependent selection (Engen et al., 2013) with age‐structured demographic models. This process has already begun with more sophisticated eco‐evolutionary density‐dependent selection models of life‐history evolution that explore the effects of optimal age of maturity (Engen & Sæther, 2016), that separate effects on birth rates versus death rates (Engen & Sæther, 2017) and that include age‐structured populations explicitly (Lande, Engen, & Sæther, 2017). However, none of these models predicts a greater variety of life histories beyond the single fast–slow axis. In order to explain the diversity of life histories we see in nature, additional and possibly orthogonal life‐history dimensions (e.g., the fast reproduction axis alongside the slow survival axis of trees and large fish) are needed beyond the simple one‐dimensional fast–slow axis. It seems reasonable to expect that such multiple dimensions of fast–slow life‐history variation can only exist if the population dynamics at different ages or life stages are sufficiently independent to allow the direction of density‐dependent selection to differ in contrasting parts of the life history, such as in offspring survival selection versus adult reproduction selection.

The aim of this paper was to extend density‐dependent selection theory in life‐history evolution developed by Lande et al. (2009, 2017), Engen et al. (2013), and Engen and Sæther (2016, 2017). We explore how density‐dependent selection can facilitate independent life‐history evolution at different life stages, as opposed to the usual unidimensional fast–slow continuum in which all life stages coevolve to match the same pace of life.

## 2 METHODS

### 2.1 The general model

We consider a large population of an organism with two life stages, adults and offspring, and nonoverlapping generations. At time *t,* there are *N_t_* adults producing *n_t_* offspring that enter the adult population in the next time step, if they survive. The dynamics is then given by the sequence *N_t_*, *n_t_*, *N_t_*_+1_, *n_t_*_+1_,…. The fecundity of an adult individual with phenotype **z** at time *t* is *F*(**z**, *N_t_*)Λ*_F_*_,_ *_t_*, where Λ*_F,t_* is an environmentally fluctuating factor with mean 1 and *F*(**z**, *N_t_*) is the density‐dependent fecundity in the average environment. We assume that **z** at time *t* has a multivariate normal distribution *p*(**z**; ${\bar{z}}_{t}$, **P**) among adult individuals with evolving mean ${\bar{z}}_{t}$ and covariance matrix **P** assumed to be constant. The mean fecundity given the environment,

(1) $$\bar{F}\left({\bar{z}}_{t},N_{t}\right)\Lambda_{F,t}=\Lambda_{F,t}\int F\left(z,N_{t}\right)p\left(z;{\bar{z}}_{t},P\right)dz,$$

then determines the number of offspring:

(2) $$n_{t}=N_{t}\bar{F}\left({\bar{z}}_{t},N_{t}\right)\Lambda_{F,t}.$$

The selection differential in the fecundity step is the difference between the mean phenotype of the offspring's parents and the mean in the parental population as a whole,

(3) $$\Delta {\bar{z}}_{F,t}=\int z\left[F\left(z,N_{t}\right)/\bar{F}\left({\bar{z}}_{t},N_{t}\right)\right]p\left(z;{\bar{z}}_{t},P\right)dz-{\bar{z}}_{t},$$

where the stochastic factor cancels out. Taking the transition into account, the mean phenotype of offspring is:

(4) $${\bar{z}}_{F,t}={\bar{z}}_{t}+{\mathrm{GP}}^{-1}\Delta {\bar{z}}_{F,t},$$

where **G** is the constant additive genetic covariance matrix. The density‐dependent offspring survival into the adult population the next time step is *S*(**z**, *n_t_*)Λ*_S,t_*, where Λ*_S,t_* is another stochastic environmental factor with mean 1, which in general may be correlated with the stochastic fecundity factor. The adult population the next time step given the environment is then:

(5) $$N_{t+1}=n_{t}\bar{S}\left({\bar{z}}_{F,t},n_{t}\right)\Lambda_{S,t},$$

where $\bar{S}\left({\bar{z}}_{F,t},n_{t}\right)$ is the mean survival of offspring before selection on survival. The mean phenotype after selection, that is, the mean phenotype of offspring at the next time step is then:

(6) $${\bar{z}}_{t+1}=\int z\left[S\left(z,n_{t}\right)/\bar{S}\left({\bar{z}}_{F,t},n_{t}\right)\right]p\left(z;{\bar{z}}_{F,t},P\right)dz.$$

Here, the stochastic factor again cancels out, but the fluctuations in the environment will affect the evolutionary process through its effect on the population sizes appearing in the fecundity and survival functions.

### 2.2 Specific assumptions

In order to perform simulations used to illustrate the results here, the model must be fully specified. We assume that log fecundity has the form:

(7) $$\ln F\left(z,\;N_{t}\right)=r_{F}\left(z\right)-\gamma_{F}\left(z\right)g\left(N_{t}\right),$$

where *r_F_*(**z**) is the growth rate of a hypothetical subpopulation of individuals with phenotype **z** from the adult to the offspring stages at small densities, where the increasing function *g*(*N_t_*) is approximately zero. The function *g*(*N_t_*) describes the type of density regulation, for example, a logistic type if the function is linear. The function γ*_F_*(**z**) expresses the effect of density regulation on fecundity.

Survival is defined in a similar way with minor adjustments required to ensure that survival takes values between zero and one, as described in the Appendix S1. The stochastic effects on fecundity and survival are assumed to be log‐normally distributed with a mean of one and specified variances of var(lnΛ*_F,t_*) = *σ* ^2^_fec_ and var(lnΛ*_S,t_*) = *σ* ^2^_sur_, respectively. Details are given in the Appendix S1.

Furthermore, we assume a two‐dimensional phenotypic vector **z** = (*z*_1_, *z*_2_), where the fecundities depend on *z*_1_ and survivals on *z*_2_, with no genetic or phenotypic covariances assumed between z_1_ and z_2_. Including only joint first and second order effects in phenotype and population sizes, we let *r_F_*(*z*_1_) and *r_S_*(*z*_2_) be second order polynomials, while the density‐dependent effects *γ_F_*(*z*_1_) and *γ_S_*(*z*_2_) are linear as they occur in products with population sizes (Sæther et al., 2016). For specific details, see Appendix S1 and below where we give all of the calculations required to perform the joint simulations of population sizes and phenotypes.

### 2.3 The simulations

In order to simulate the evolution of the two phenotypes for adult reproduction (${\bar{z}}_{1}$) and offspring survival (${\bar{z}}_{2}$) under different population dynamics and environmental conditions, it is necessary to use some of the Equations ((1), (2), (3), (4), (5), (6)) above and in Appendix S1. Here, we provide details of how to do this for a given set of parameter values. First, the initial values for *N*_0_ and ${\bar{z}}_{0}={\left[{\bar{z}}_{1,0},{\bar{z}}_{2,0}\right]}^{T}$ need to be generated. Then, for each time step *t* from 0 to *t*_max_ one needs to:

- Draw *ε_F,t_* ~ *N*(0, 1);
- Compute *n*_t_ using Equation (S17) in Equation (2) above;
- Compute the mean phenotype after the fecundity step ${\bar{z}}_{F,t}$ using Equation (S19);
- Draw *ε_S,t_* ~ *N*(0, 1);
- Compute *N*_t+1_ using Equation (S23) in Equation (5) above;
- Compute the mean phenotype the next time step using Equation (S32) in ${\bar{z}}_{t+1}={\bar{z}}_{F,t}+\Delta {\bar{z}}_{S,t}$;

where *ε_F,t_* and *ε_S,t_* are standard normally distributed, *N*(0, 1), so that the expectations of these factors are equal to one.

### 2.4 Model context

This model separates the life history into two life stages, within which eco‐evolutionary processes can occur that shape adult reproduction and offspring survival. It therefore also captures the contrast between life‐history trade‐offs that could be seen to occur within versus between life stages. This is because it involves the reproduction versus survival trade‐off in the form of adult reproduction selection and the offspring quality versus quantity trade‐off in terms of offspring survival selection.

To illustrate the different types of life histories produced by our model, we employ four simplistic labels: “housefly,” “blue whale,” “oak tree,” and “mayfly.” These example life histories are used as general archetypes for the purposes of illustrating the four extremes of life‐history variation. Hence, houseflies (*Musca domestica*, like many small insects) have classical “fast” density‐independent life histories with short life spans, high rates of reproduction, and limited somatic growth or parental investment. In contrast, blue whales (*Balaenoptera musculus*, like most large mammals) have classical “slow” density‐dependent life histories with long life spans and extensive somatic growth and investment, a late age at first reproduction and extensive parental investment per offspring. Oak trees (*Quercus* spp. and indeed any large trees) seem at first sight to be the plant equivalent of slow‐selected life histories of blue whales, at least in their slow growth and long‐lived adult life stage with extreme iteroparity, probably as a result of strong density dependence on oak tree numbers within a woodland canopy. However, the large numbers of offspring produced by oak trees with limited parental investment per offspring suggests a relatively fast‐selected offspring life history, with limited density dependence on the numbers of acorns that survive on the woodland floor to germinate. Mayflies (Ephemeroptera) perhaps represent an example of the last of these four archetypes, because we can think of short‐lived reproductive lifespan of the small flying adults as fast‐selected and largely independent of the density of others involved in the same mating flight. However, the growth and survival of much longer‐lived and larger offspring in the form of nymphs might represent a relatively slower life stage that tends to be under strong density‐dependent selection. Possible descriptive inaccuracies and empirical exceptions notwithstanding (see the discussion below) these animal names simply serve to provide convenient labels with which to refer to the four extreme life‐history outcomes of our model.

## 3 RESULTS

**Figure 1:**
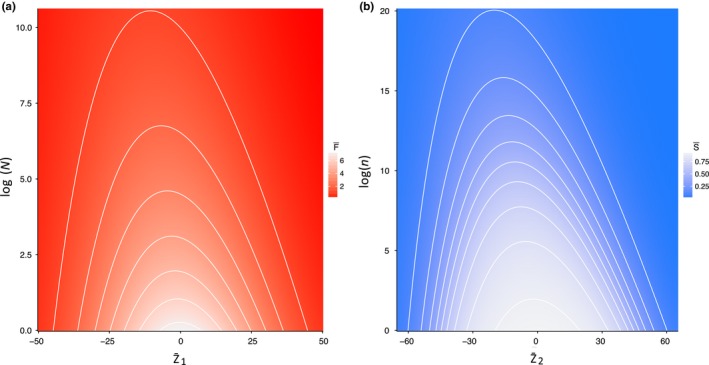
The effect of population density on fitness contours (higher values indicated by lighter colors) for different values of a general population mean life‐history $\bar{z}$ (arbitrarily higher trait values indicate density‐independent selection, while lower trait values indicate density‐dependent selection). Greater fitness is achieved by evolving a lower value of $\bar{z}$ when at higher population densities for both (a) the effect of adult population density log(*N*) and the mean adult reproduction trait (${\bar{z}}_{1}$) on reproductive fitness ($\bar{F}$), and (b) the effect of offspring population density log(*n*) and the mean offspring survival to adulthood trait (${\bar{z}}_{2}$) on survival fitness ($\bar{S}$). Parameter values for adult reproduction: *α*_0_ = 2, *α*_1_ = 0.001, *α*_2_ = 0.2, *α*_3_ = 0.002, and *z_α_* = 0; and for offspring survival: *β*_0_ = 2, *β*_1_ = 0.001, *β*_2_ = 0.2, *β*_3_ = 0.002, and *z_β_ *= 0—see Appendix S1 for details

Each of the two life stages within our extended model replicates the main analytical result of Engen et al. (2013) showing the eco‐evolutionary dynamics of density‐independent versus density‐dependent selection. As population sizes increase toward the carrying capacity *K*, the mean population value of the life‐history trait $\bar{z}$ changes (in our case decreases). This is similarly true of the adult reproductive population (*N*) and mean adult reproduction (${\bar{z}}_{1}$) maximizing reproductive fitness ($\bar{F}$), and of the offspring population (*n*) and mean offspring survival (${\bar{z}}_{2}$) maximizing survival fitness ($\bar{S}$)—see Figure 1. Hence, variation in density‐independent versus density‐dependent selection can be produced in both adult reproductive and offspring survival strategies.

**Figure 2:**
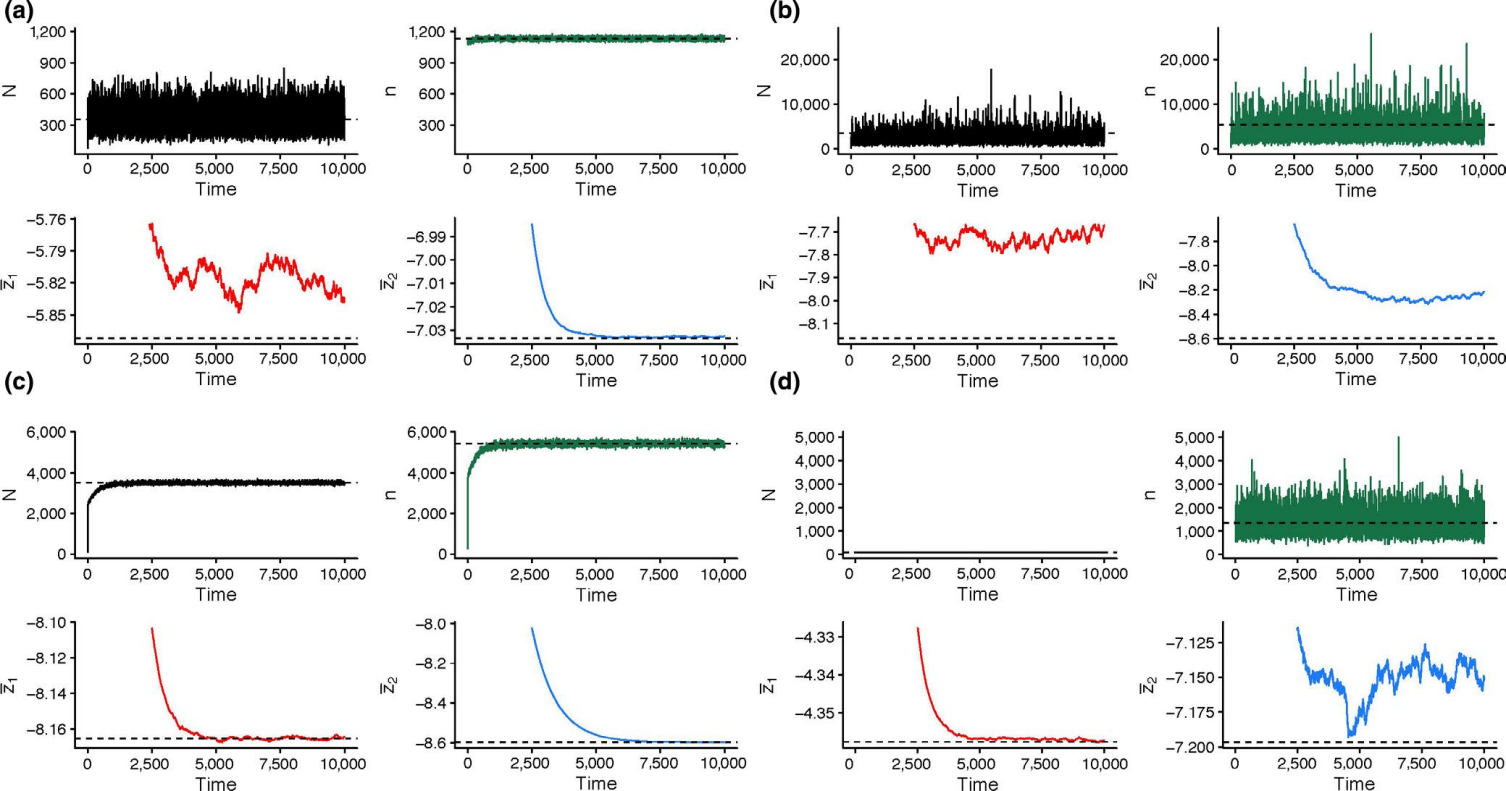
Examples of model simulation results representing the four extreme types of life history: (a) “mayflies” with fast‐selected adult reproduction and slow‐selected offspring survival; (b) “houseflies” with fast‐selected reproduction and survival; (c) “blue whales” with slow‐selected reproduction and survival; and (d) “oak trees” with slow‐selected reproduction and fast‐selected survival. In each panel, changes over time (generations) are shown as lines for populations sizes of adults (*N*, black) and offspring (*n*, green), and for values of the reproduction (${\bar{z}}_{1}$, red) and survival (${\bar{z}}_{2}$, blue) traits, with deterministic values for simulations with no environmental stochasticity (black dashed horizontal lines) for comparison. Parameter values for (a): *α*_0_ = 7, *α*_1_ = 0.001, *α*_2_ = 1.0, *α*_3_ = 0.002, and *z_α_* = 0; and *β*_0_ = 3, *β*_1_ = 0.001, *β*_2_ = 0.5, *β*_3_ = 0.002, and *z_β _*= 0; and *σ* ^2^_fec_ = 0.0001 and *σ* ^2^_sur_ = 0.1. For B and C: *α*_0_ = 2, *α*_1_ = 0.001, *α*_2_ = 0.2, *α*_3_ = 0.002, and *zα* = 0; and *β*_0_ = 2, *β*_1_ = 0.001, *β*_2_ = 0.2, *β*_3_ = 0.002, and *z_β_* = 0. In (b), *σ* ^2^_fec_ = 0.1 and in survival *σ* ^2^_sur_ = 0.1, while in (c) *σ* ^2^_fec_ = 0.0001 and in survival *σ* ^2^_sur_ = 0.0001. For (d): *α*_0_ = 5, *α*_1_ = 0.001, *α*_2_ = 0.5, *α*_3_ = 0.002, and *zα* = 0; and *β*_0_ = 5, *β*_1_ = 0.001, *β*_2_ = 1.0, *β*_3_ = 0.002, and *z_β_* = 0; with *σ* ^2^_fec_ = 0.1 and in survival *σ* ^2^_sur_ = 0.0001. In all four cases, *σ*_1_ = 1, *σ*_2_ = 1, *ρ *= 0, *G*_11_ = 1, *G*_12_ = 0, *G*_21_ = 0, and *G*_22_ = 1. See text, Section 2 and Appendix S1 for further details

Simulation results demonstrate that the extent of this selection on fast versus slow life histories in the two life stages is crucially dependent upon the strength of density dependence on adult reproduction and/or offspring survival. As Figure 2 illustrates, without strong density dependence (i.e., *α*_2_ < 0.75, *β*_2_ < 0.75) any stochastic variation introduced into adult population sizes (*N*) by the environment carries over as associated stochastic variation in offspring population sizes (*n*), and *vice‐versa* with stochasticity introduced into *n* carrying over as associated stochasticity in *N*. Thus, with limited density dependence, increasing stochastic variation in either *N* or *n*, or both (i.e., *σ* ^2^_fec_ = 0.0001–0.1 and *σ* ^2^_sur_ = 0.0001–0.1), modifies the overall life history from slow‐selected “blue whale” (Figure 2c) to fast‐selected “housefly” (Figure 2b), creating a positive covariance between trait values for adult reproduction (${\bar{z}}_{1}$) and offspring survival (${\bar{z}}_{2}$).

However, even with considerable stochastic variation in fecundity (*σ* ^2^_fec_ = 0.1) and thus also in *n*, strong density dependence on the number of those offspring that survive to become reproductive adults (*β*_2_ = 1.0) allows slow selection on adult reproduction ${\bar{z}}_{1}$ to exist separately from the fast selection on offspring survival ${\bar{z}}_{2}$ (an “oak tree,” Figure 2d). Conversely, despite substantial stochasticity in the number of offspring that become reproductive adults (*σ* ^2^_sur_ = 0.1) and thus also in *N*, strong density dependence on the number of offspring produced (*α*_2_ = 1.0) allows slow selection in ${\bar{z}}_{2}$ separate from the fast selection in ${\bar{z}}_{1}$ (a “mayfly,” Figure 2a).

**Figure 3:**
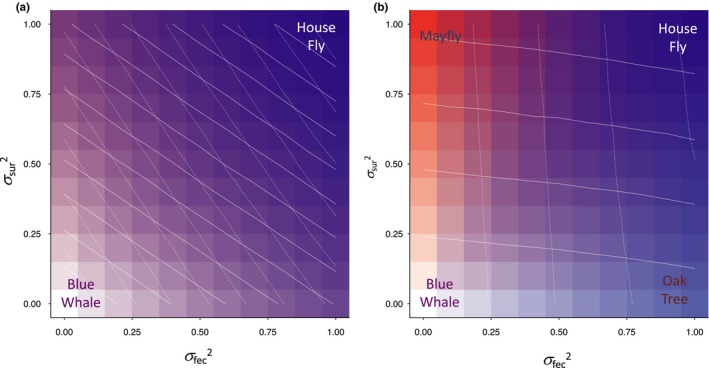
Model simulation results for evolved values for the adult reproductive life‐history trait ${\bar{z}}_{1}$ (red) and offspring survival life‐history trait ${\bar{z}}_{2}$ (blue), relative to deterministic values with no environmental stochasticity (white). Darker colors indicate higher (i.e., greater fast selection) relative mean trait values, while purple indicates a mix of red and blue. White contours further indicate 0.3 interval changes in these mean trait values (${\bar{z}}_{1}$ solid contours, ${\bar{z}}_{2}$ dashed contours). Results are shown for increasing levels of environmental stochasticity in offspring survival populations (*σ* ^2^_sur_) and in adult reproductive populations (*σ* ^2^_fec_), causing increasing levels of density‐independent selection in the evolved values of ${\bar{z}}_{1}$ and ${\bar{z}}_{2}$, respectively. In (a), limited density dependence of adult (*N*) and offspring (*n*) populations (i.e., *α*_2_ = 0.5, *β*_2_ = 0.5) allows stochasticity in the population dynamics at one life stage to carry over into the other. This produces the traditional one‐dimensional fast to slow life‐history continuum (from a “housefly” to a “blue whale”) via simultaneous changes in the contours of ${\bar{z}}_{1}$ and ${\bar{z}}_{2}$diagonally down from purple to white (top right to bottom left). However, in (b) strong density dependence in both *N* and *n* (i.e., *α*_2_ = 1, *β*_2_ = 1) allows quasi‐independent population dynamics in the two life stages. This results in orthogonal changes in the contours in ${\bar{z}}_{1}$ and ${\bar{z}}_{2}$, producing in the extreme corners all four colors and alternative types of life history (“housefly,” “blue whale,” “oak tree,” and “mayfly”). In (b), the simulation dynamics in the four corners are qualitatively similar to those shown in Figure 2. Results shown are mean values of 100 simulations after 1,000 generations, with other parameter values: *α*_0_ = 7, *α*_1_ = 0.001, *α*_3_ = 0.005; *β*_0_ = 5, *β*_1_ = 0.001, *β*_3_ = 0.005; and *σ*_1_ = 1, *σ*_2_ = 1, *ρ* = 0, *G*_11_ = 1, *G*_12_ = 0, *G*_21_ = 0, and *G*_22_ = 1. See Section 2 and Appendix S2 for further details

Figure 3 shows these simulation results for more standardized parameter values across the full range of values of stochastic variation (i.e., *σ* ^2^_fec_ = 0.0 to 1.0 and *σ* ^2^_sur_ = 0.0 to 1.0) in the adult population (*N*) versus the offspring population (*n*). Increasing stochastic variation in population sizes in both life stages simultaneously leads to the classic one‐dimensional life‐history axis from slow selection to fast selection (“blue whale” bottom left to “housefly” top right in Figure 3a). However, as Figure 3b shows, strong density dependence in both life stages (i.e., *α*_2_ = 1, *β*_2_ = 1) allows the evolution of all four life‐history strategies (“housefly,” “blue whale,” “oak tree,” and “mayfly”), due to independent evolution that is now possible in the traits for adult reproduction (${\bar{z}}_{1}$) versus offspring survival (${\bar{z}}_{2}$).

## 4 DISCUSSION

Our eco‐evolutionary model demonstrates that separating out the strength of density dependence at different life stages has the potential to explain much of the life‐history variation found among species in nature. Strong density dependence on either adult reproduction and/or offspring survival can create contrasting population dynamics at different life stages in terms of the degree to which environmental stochasticity reduces the mean population densities experienced by adults versus offspring. This allows the possibility of slow‐selected adult reproduction alongside fast‐selected offspring survival (e.g., oak trees) or fast‐selected reproduction alongside slow‐selected survival (e.g., mayflies). However, in most cases, insufficiently strong density dependence at either life stage probably causes the carryover of environmental stochasticity in population sizes from one part of the life history to the other(s). Hence, even with this separation into different life stages, for most species the degree of density‐dependent selection may end up being similar in both adult reproduction and offspring survival, resulting in the traditional one‐dimensional fast–slow continuum of houseflies to blue whales, as seen in most organisms—see Section 1.

Therefore, our main finding is that strong density dependence can isolate any effects of environmental stochasticity within each of the different age classes or life stages, which can potentially produce additional axes of fast‐ versus slow‐selected life‐history variation. For many species on the usual one‐dimensional fast–slow continuum, such as our archetypal slow‐selected blue whales, the same density‐dependent effects of intraspecific competition should affect both juvenile survival and adult reproduction, because juveniles and adults interact as part of the same population. Indeed, in such cases we do not necessarily need a model with two life stages, and the original Engen et al. (2013) model with its single population and single life‐history trait would suffice. The same might also be true for our archetypal fast‐selected houseflies, although the existence of a larval life stage in many (winged) insect taxa suggests an ecological separation of larval versus adult population dynamics. Since most insect life histories show apparently fast‐selected traits at both larval and adult life stages, this could be the result of fast selection at either life stage with weak density dependence and carryover effects of environmental stochasticity on population densities within cohorts of larvae and adults (as in Figure 3a), or alternatively fast selection might be occurring separately within each life stage (as in Figure 3b).

For species with more complex life histories that deviate from the traditional fast–slow continuum, we predict quasi‐independent population dynamics at different life stages due to strong density dependence in one or more of those life stages. For example, adult reproduction in oak trees, with its intense intraspecific competition for light in the canopy and for water and nutrients for roots deep in the soil, is almost certainly subject to strong density dependence, as compared with acorn survival with its density‐independent probabilities of predation versus successful germination on the woodland floor (see Keator, 1998). Likewise, large teleost fish are often spatially if not ecologically separated from their larval and juvenile young, and in the case of strong density dependence the eco‐evolutionary dynamics of life‐history evolution must occur as part of two (or more) quasi‐independent processes at the different life stages (see Winemiller, 2005; Wootton, 1990). It should be noted here that it is possible to conceive of more complex models of the type presented here with more than just two life stages. As long as there is sufficient independence in the separate population dynamics at these different life stages (e.g., fish eggs, larvae, juveniles, and adults could all exist in sufficiently separated environments), then such models would generate even more complex life histories that would then require quantification/classification using three or more axes of fast–slow life‐history variation.

**Figure 4:**
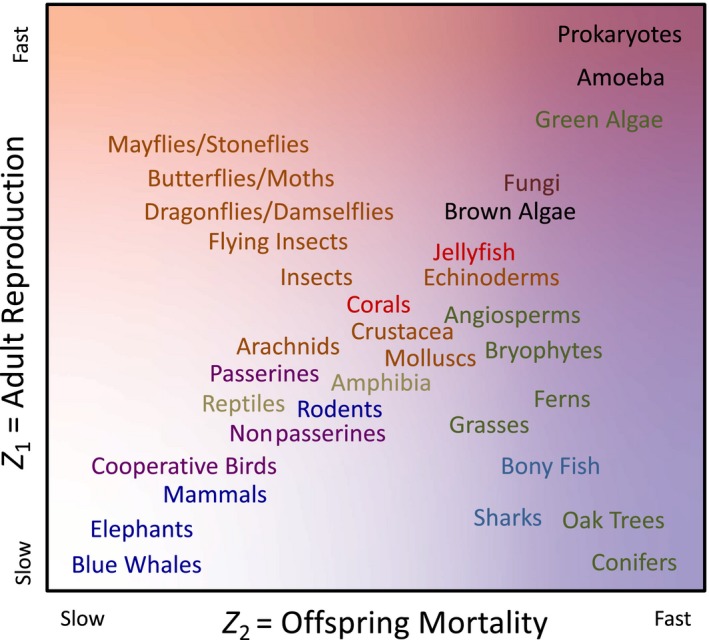
Illustration of the possible positions of different taxonomic groups in a notional two‐dimensional continuum from fast‐selected to slow‐selected life histories in adult reproduction (red, equivalent to our trait ${\bar{z}}_{1}$) and offspring mortality (blue, equivalent to our trait ${\bar{z}}_{2}$). The main axis of variation remains bottom left (slow‐selected “blue whales”) to top right (fast‐selected micro‐organisms beyond “houseflies”), as in Figure 3a. However, an additional second axis of variation could be seen to exist for some taxonomic groups, bottom right (slow‐selected reproduction and fast‐selected offspring mortality “oak trees”) to top left (fast‐selected reproduction and slow‐selected offspring mortality “mayflies”), as in Figure 3b. The colored background from white to red to blue to purple indicates the relative values of the two traits, as in Figure 3b

Regarding our last archetypal life history, the “mayfly” in the top left of Figures 2 and 3, we are thinking here of various types of winged insects with population densities determined mostly by survival of slow growing juveniles and mating flights by relatively short‐lived adults (e.g., Plecoptera and Ephemeroptera). It is interesting to note that outside of these groups it is difficult to think of many more examples of taxa with fast selection on adult reproduction and slow selection on offspring survival. To illustrate this, Figure 4 provides a speculative arrangement of life histories for different taxonomic groups, based upon variation in the two traits in our model. As we explain in the Introduction, most taxa align diagonally along the traditional fast–slow continuum (top right to bottom left of Figure 4, as in Figure 3a). We can also think of a number of taxa, such as large fish and trees, that extend into the bottom right corner of Figure 4, with slow selection on adult reproduction and fast selection on offspring survival (Figures 2d and 3b). However, this second life‐history axis predicted by our model, running top left to bottom right in Figure 4, does not seem to have too many obvious examples that we could place in the top left corner (Figures 2a and 3b), even if a case could be made for certain flying insects with mating flights such as mayflies, stoneflies, and perhaps some species of butterflies and moths (Lepidoptera), dragonflies and damselflies (Odonata), and cicadas (Cicadidae). It is important to note that the value of Figure 4 is purely heuristic at this stage, and it is shown here simply as a stimulus to further thought and debate as to the relative positions of the different taxa. It is an interesting question how many taxonomic groups within the insects, and therefore how many species in total, might reasonably be placed at some point along the axis extending toward to top left corner. Hopefully, our model predictions will encourage more detailed empirical research into the effects of life stage‐specific demographics on life‐history variation.

Our model results therefore include the possibility of a whole class of life histories that may not often exist in nature. It is interesting to ask why this is, because perhaps there is some kind of constraint or costly trade‐off is missing from our model, and perhaps from density‐dependent life‐history theory in general. Most obviously, the combination of fast selection on adult reproduction and slow selection on offspring survival may be rare in nature because it requires extreme density dependence on the number of offspring, such that no amount of variation in the size of the adult population feeds through to affect variation in the size of the offspring population (Figure 2a). Given the smaller physical size of offspring compared with adults in most systems, it is perhaps rare that offspring can ever be so much more resource limited than their parents so as to nullify any effect of variation in adult numbers on offspring population sizes. Hence, while it is theoretically possible in our model, examples of such extreme life histories may have rarely, if ever, evolved because of the basic physical constraint of directional somatic growth that causes increasing individual resource demands with age in the vast majority of taxa. Our contention is that certain winged insects, such as Lepidoptera or Ephemeroptera, may provide possible examples here, if there is density‐dependent survival during the long‐lived large larval/juvenile life stage and any short‐lived small‐bodied adult life stage is dedicated to reproduction that is largely density‐independent. This therefore suggests a need for further investigation informed by our model predictions concerning the nature of density‐independent versus density‐dependent selection in taxa with metamorphosis or similar processes and ecologically distinct juvenile versus adult life stages.

It should be remembered that although our model, like that of Engen et al. (2013), is couched in terms of environmental stochasticity limiting population sizes below carrying capacity, it is perfectly possible for demographic stochasticity to produce qualitatively similar effects on life‐history evolution. Hence, fast‐selected life histories can be generated in populations limited by high levels of demographic stochasticity, for example in the form of random predation, where there is an individual probability of predation that is largely density‐independent and unpredictable and cannot be adaptively reduced further through the evolution of antipredator traits. The only difference is that such demographic stochasticity (via effects such as individual predation probabilities) will not have the same effects as environmental stochasticity (via such things as weather affecting whole populations) on variances in fitness that can lead to the evolution of additional bet‐hedging adaptations in life‐history strategies (see Wright et al., 2019). Likewise, any density‐dependent effects in models like this are perhaps usually imagined in terms of intraspecific competition for the resources needed to survive and reproduce. However, since such mechanisms are technically unspecified, they could instead be driven by positively density‐dependent rates of predation at different life stages. Our fully eco‐evolutionary framework for the understanding the (co)evolution of life‐history traits at different life stages can therefore easily be extended to include predation in many of these same demographic processes.

The crucial point here is that life‐history evolution can only be understood through its interaction with population dynamics and specifically the role of density‐dependent effects in limiting population sizes. Indeed, apparent problems in many earlier life‐history studies can perhaps be traced back to a failure to properly appreciate the eco‐evolutionary feedbacks that are a critical part of density‐independent versus density‐dependent selection. For example, early laboratory selection experiments on protozoa, *Drosophila* spp., and other small invertebrates failed to show consistent evolutionary effects on life histories of maintaining populations at carrying capacity versus at densities well below carrying capacity (e.g., Luckinbil, 1979; Taylor & Condra, 1980; Barclay & Gregory, 1981; Mueller & Ayala, 1981; Bergmans, 1984; see reviews in Bradshaw & Holzapfel, 1989; Reznick et al., 2002), and this has been interpreted as another piece of evidence against *r*‐ versus *K*‐selection theory (Stearns, 1993). However, the selective conditions in these types of experiments tend to be exclusive to the particular laboratory environment, precipitating the evolution of particular traits that may have had little connection to the natural eco‐evolutionary dynamics of the species within which the life‐history evolved. When the natural source of any population limitation has been identified, and that source is artificially manipulated, then the full eco‐evolutionary feedback of density‐dependent selection should be revealed as part of any effect of artificial selection on the life history. A recent example of this is in the experimental work of Reznick et al. (2019), involving the well‐studied populations of guppies in different streams on Trinidad, which experience contrasting levels of predator‐mediated population limitation. By manipulating the presence or absence of specific types of predators to control the densities of experimental populations, Reznick et al. (2019) induced genetically based changes in density‐dependent selection on guppy life histories that matched those of the natural populations. Time lags in the appearance of these experimental effects were specifically interpreted as indicative of eco‐evolutionary feedbacks, as we might expect from density‐independent versus density‐dependent selection. It is only through this kind of appreciation of natural sources of population limitation and the eco‐evolutionary feedbacks involved that can we properly study and understand life‐history evolution.

Our model continues the development of a modern eco‐evolutionary density‐dependent selection theory started by Lande et al. (2009) and Engen et al. (2013). It provides a much‐needed mathematical framework for the evolution of specific life‐history traits, which was one of the main criticisms of MacArthur and Wilson's (1967) original *r*‐ versus *K*‐selection theory (Charlesworth, 1980; Stearns, 1993). We can therefore predict the role of any trait in driving density‐dependent life‐history evolution through its effects on the parameters describing density‐independent reproduction (*r*_0_) and density‐dependent effects on fitness (*γ*) estimated from natural populations (e.g., Sæther et al., 2016). In principle, these same effects can be investigated across multiple traits simultaneously using multivariate statistical analyses, such as structural equation modeling, at the species, population, and/or individual levels (Wright et al., 2019). Indeed, there is a clear connection here between fast–slow life‐history variation among species and fast–slow “pace‐of‐life syndromes” (POLS) among individuals within populations. Wright et al. (2019) have recently argued that with stochastic environmental variation and thus under fluctuating density‐dependent selection, then the same Engen et al. (2013) model framework used here predicts that any fast–slow life‐history variation among species will be mirrored (i.e., along the same orientation of fast–slow multi‐trait axis) in fast versus slow life histories among individual in POLSs seen within each of these species or populations. Hence, our model raises the additional possibility of one than one axis of life‐history variation in POLSs within populations, if the population dynamics of the different life stages are sufficiently independent of each other. Current theoretical models also show how this framework can be developed to explore how the parameters *r*_0_ and *γ* are shaped by changes in the optimal age of maturity (Engen & Sæther, 2016), by changes in birth rates versus death rates (Engen & Sæther, 2017) and by selection varying at different ages (Lande et al., 2017). Our model demonstrates the crucial possibility of more than one axis of fast versus slow selection on life‐history variation, and thus how different sets of values for *r*_0_ and *γ* may exist at different life stages within the same system. Such a situation thus requires further statistical decomposition to quantify the effects of specific traits on the values of *r*_0_ and *γ* within each life stage; for example, as part of the reproduction versus survival trade‐off in adult reproduction selection and/or the trade‐off between the quality versus quantity of offspring in offspring survival selection. Our results therefore suggest that an understanding of population regulation at different ages and/or life stages is critical if we are to correctly predict and understand the role of specific traits in the life history of a particular species.

In conclusion, perhaps some crucial ideas in *r*‐ versus *K*‐selection theory have been prematurely rejected (see also Boyce, 1984). Indeed, many of these ideas still persist rather vaguely in the current literature in terms of the fast–slow pace‐of‐life continuum. Either way, we have for too long lacked an appropriately rigorous theoretical eco‐evolutionary approach to life‐history variation. This has now been provided by the development of modern density‐dependent selection theory (Engen et al., 2013; Engen & Sæther, 2016, 2017; Lande et al., 2009, 2017), including the ideas we develop here. We hope that this will open up further opportunities for more structured explorations of life‐history evolution in more realistic ecological detail. For example, statistical analyses centered upon the demographic consequences of individual life‐history traits in terms of their contributions to density‐independent reproductive rates (*r*_0_) and density‐dependent effects on fitness (*γ*) (Sæther et al., 2016; Wright et al., 2019). In addition, we need more artificial density‐dependent selection experiments along the lines of Reznick et al. (2019) that manipulate natural sources of population limitation, whether they involve environmental and/or demographic stochasticity. Hopefully, our model provides a number of original testable predictions and avenues for future research on life‐history evolution, including its role in mediating natural population responses to environmental change.

## CONFLICTS OF INTEREST

The authors declare no conflicts of interest.

## AUTHOR CONTRIBUTIONS

JW conceived the original idea and drafted the manuscript. SE produced the mathematical model and supervised the simulations. EBS created and ran the simulations, including Figures 1, 2, 3, and wrote the Appendix. All authors contributed to the final manuscript text and gave final approval for publication.

## Data Availability

Details for creating the simulations in Figures 2 and 3 are available in the Appendix.
