# Supplementary material for: Contrasting patterns of density‐dependent selection at different life stages can create more than one fast–slow axis of life‐history variation
Source: Ecol Evol. 2020 Mar 1;10(6):3068–78. doi: 10.1002/ece3.6122 (PMC7083673; doi:10.1002/ece3.6122)
Supplement: Supplementary file 1 [file ECE3-10-3068-s001.pdf]

Appendix for: 'Contrasting patterns of density-dependent selection at different life stages can create more than one fast-slow axis of life-history variation'

S1: Specific model assumptions

We use the following fecundity function

$$F(\mathbf{z}, N_t) = e^{r_F(\mathbf{z}) - \gamma_F(\mathbf{z}) g(N_t)} \tag{S1}$$

where $r_F(\mathbf{z})$ is the density independent deterministic growth rate of the fecundity, while $\gamma_F(\mathbf{z})$ is the strength of density regulation and $g(\cdot)$ describes the shape of the density regulation. While the range of $F(\mathbf{z}, N_t)$ is $[0, \infty)$, the survival function has to be between zero and one, so we assume the following surival function

$$S(\mathbf{z}, n_t) = \begin{cases} \frac{1}{2} e^{r_S(\mathbf{z}) - \gamma_S(\mathbf{z}) g(n_t)} & \text{if} \quad r_S(\mathbf{z}) - \gamma_S(\mathbf{z}) g(n_t) < 0 \\ 1 - \frac{1}{2} e^{-(r_S(\mathbf{z}) - \gamma_S(\mathbf{z}) g(n_t)} & \text{if} \quad r_S(\mathbf{z}) - \gamma_S(\mathbf{z}) g(n_t) \geq 0 \end{cases} \tag{S2}$$

where $r_S(\mathbf{z})$ is the growth rate and $\gamma_S(\mathbf{z})$ is the strength of density regulation of the survival. Other choices of survival function includes the logistic function or probit function, but the function above is similar in shape and we can compute the results exact, without relying on any numerical approximations of the integration.

The environmental factors in fecundity and survival are

$$\Lambda_{F,t} = e^{-\sigma^2_{\text{fec}}/2 + \sigma_{\text{fec}} \varepsilon_{F,t}} \tag{S3}$$

$$\Lambda_{S,t} = e^{-\sigma^2_{\text{sur}}/2 + \sigma_{\text{sur}} \varepsilon_{S,t}} \tag{S4}$$

where $\varepsilon_{F,t}$ and $\varepsilon_{S,t}$ are standard normally distributed, $\mathcal{N}(0, 1)$, so the expectations of the factors are equal to one. For a given value of $\Lambda_{F,t}$, we can write

$$F(\mathbf{z}, N_t)\Lambda_{F,t} = e^{r_F(\mathbf{z}) - \gamma_F(\mathbf{z}) g(N_t) + \ln \Lambda_{F,t}} \tag{S5}$$

and similarly, for a given value of $\Lambda_{S,t}$, the survival is

$$S(\mathbf{z}, n_t)\Lambda_{S,t} = \begin{cases} \frac{1}{2} e^{r_S(\mathbf{z}) - \gamma_S(\mathbf{z}) g(n_t) + \ln \Lambda_{S,t}} & \text{if} \quad r_S(\mathbf{z}) - \gamma_S(\mathbf{z}) g(n_t) + \ln \Lambda_{S,t} < 0 \\ 1 - \frac{1}{2} e^{-(r_S(\mathbf{z}) - \gamma_S(\mathbf{z}) g(n_t) + \ln \Lambda_{S,t})} & \text{if} \quad r_S(\mathbf{z}) - \gamma_S(\mathbf{z}) g(n_t) + \ln \Lambda_{S,t} \geq 0 \end{cases} \tag{S6}$$

In our analysis we have assumed that we only have two phenotypes, so $\mathbf{z} = [z_1, z_2]^T$ has a bivariate normal distribution:

$$p(\mathbf{z}; \bar{\mathbf{z}}, \mathbf{P}) = \frac{1}{2\pi\sigma_1\sigma_2\sqrt{1-\rho^2}} \exp\left\{ -\frac{1}{2(1-\rho^2)} \left[\frac{(z_1 - \bar{z}_1)^2}{\sigma_1^2} - \frac{2\rho(z_1 - \bar{z}_1)(z_2 - \bar{z}_2)}{\sigma_1\sigma_2} + \frac{(z_2 - \bar{z}_2)^2}{\sigma_2^2} \right] \right\} \tag{S7}$$

where $\bar{\mathbf{z}} = [\bar{z}_1, \bar{z}_2]^T$ and $\mathbf{P}$ is a 2×2 matrix with rows equal to $\mathbf{P}_{1\cdot} = [\sigma_1^2, \rho\sigma_1\sigma_2]$ and $\mathbf{P}_{2\cdot} = [\rho\sigma_1\sigma_2, \sigma_2^2]$. Furthermore, we assume that the growth rates and strengths of density dependence have the following shapes

$$r_F(\mathbf{z}) = \alpha_0 - \alpha_1(z_1 - z_\alpha)^2 \tag{S8}$$
$$\gamma_F(\mathbf{z}) = \alpha_2 + \alpha_3 z_1 \tag{S9}$$
$$g(\cdot) = \ln(\cdot) \tag{S10}$$
$$r_S(\mathbf{z}) = \beta_0 - \beta_1(z_2 - z_\beta)^2 \tag{S11}$$
$$\gamma_S(\mathbf{z}) = \beta_2 + \beta_3 z_2 \tag{S12}$$

Fecundity is only a function of phenotype z_1, while survival is a function of z_2. The shape of density regulation is of the Gompertz type (linear on log scale). Since we have assumed that fecundity and survival are functions of a single phenotype, z_1 and z_2 respectively, we can integrate out the other and are left with the marginal distribution of $z_1 \sim \mathcal{N}(\bar{z}_1, \sigma_1^2)$ or $z_2 \sim \mathcal{N}(\bar{z}_2, \sigma_2^2)$. The following equation will be usefull:

$$\int e^{ax^2+bx+c}\mathcal{N}(x;\mu,\sigma^2)dx = \frac{1}{\sqrt{1-2\sigma^2 a}}\exp\left\{\frac{a\mu^2+b\mu+b^2\sigma^2/2}{1-2\sigma^2 a}+c\right\}\int \mathcal{N}\left(x;\frac{\mu+\sigma^2 b}{1-2\sigma^2 a},\frac{\sigma^2}{1-2\sigma^2 a}\right)dx. \tag{S13}$$

For a given environment, we can compute the mean fecundity $\bar{F}(\bar{\mathbf{z}}_t, N_t)\Lambda_{F,t}$ using Equation (S13) where

$$a = -\alpha_1 \tag{S14}$$
$$b = 2\alpha_1 z_\alpha - \alpha_3 \ln N_t \tag{S15}$$
$$c = \alpha_0 - \alpha_1^2 - \alpha_2 \ln N_t + \ln \Lambda_{F,t} \tag{S16}$$

and we get

$$\bar{F}(\bar{\mathbf{z}}_t, N_t)\Lambda_{F,t} = \frac{1}{\sqrt{1+2\alpha_1\sigma_1^2}}\exp\left\{\frac{-\alpha_1\bar{z}_1^2+(2\alpha_1 z_\alpha-\alpha_3\ln N_t)\bar{z}_1+(2\alpha_1 z_\alpha-\alpha_3\ln N_t)^2\sigma_1^2/2}{1+2\alpha_1\sigma_1^2} +\alpha_0-\alpha_1 z_\alpha^2-\alpha_2\ln N_t+\ln\Lambda_{F,t}\right\} \tag{S17}$$

To find the selection differential $\Delta\bar{\mathbf{z}}_{F,t}$ using Equation (3) in the main text, we see that the nominator is $\bar{F}(\bar{z}_1, N_t)$ times the mean of the normal distribution in Equation (S13), so we get

$$\Delta\bar{\mathbf{z}}_{F,t} = \left[-\frac{\sigma_1^2}{1+2\alpha_1\sigma_1^2}(2\alpha_1(\bar{z}_{1,t}-z_\alpha)+\alpha_3\ln N_t), 0\right]^T \tag{S18}$$

We define $\mathbf{G}$ as a 2×2 matrix with rows equal to $\mathbf{G}_{1\cdot} = [G_{11}, G_{12}]$ and $\mathbf{G}_{2\cdot} = [G_{12}, G_{22}]$ ($\mathbf{G}$ is symmetric), so we have

$$\bar{\mathbf{z}}_{F,t} = \begin{bmatrix} \bar{z}_{1,t} - \frac{1}{1-\rho^2}\left(G_{11} - \rho\frac{\sigma_1}{\sigma_2}G_{12}\right)\frac{2\alpha_1(\bar{z}_{1,t}-z_\alpha)+\alpha_3\ln N_t}{1+2\alpha_1\sigma_1^2} \\ \bar{z}_{2,t} - \frac{1}{1-\rho^2}\left(G_{12} - \rho\frac{\sigma_1}{\sigma_2}G_{22}\right)\frac{2\alpha_1(\bar{z}_{1,t}-z_\alpha)+\alpha_3\ln N_t}{1+2\alpha_1\sigma_1^2} \end{bmatrix} \tag{S19}$$

Similar to the mean fecundity, we find the mean survival for a given evnironment $\Lambda_{S,t}$ using Equation (S13). For clarity, we suppress the time notation t, so that $\bar{z}_{F,t} = [\bar{z}_{1,F}, \bar{z}_{2,F}]^T$. We now have

$$a = -\beta_1 \tag{S20}$$
$$b = 2\beta_1 z_\beta - \beta_3 \ln n_t \tag{S21}$$
$$c = \beta_0 - \beta_1 z_\beta^2 - \beta_2 \ln n_t + \ln \Lambda_{S,t} \tag{S22}$$

and get

$$\bar{S}(\bar{\mathbf{z}}_{F,t}, n_t)\Lambda_{S,t} = \begin{cases} \frac{1}{2}C_{(+)} & \text{if} \quad b^2 - 4ac < 0 \\ \frac{1}{2}C_{(+)}\left[1 - \Phi\left(\frac{\bar{z}_{2,u}-\bar{z}_{2,(+)}}{\sigma_{2,(+)}}\right) + \Phi\left(\frac{\bar{z}_{2,l}-\bar{z}_{2,(+)}}{\sigma_{2,(+)}}\right)\right] & \\ \quad + \left[\Phi\left(\frac{\bar{z}_{2,u}-\bar{z}_{2,F}}{\sigma_2}\right) - \Phi\left(\frac{\bar{z}_{2,l}-\bar{z}_{2,F}}{\sigma_2}\right)\right] & \\ \quad -\frac{1}{2}C_{(-)}\left[\Phi\left(\frac{\bar{z}_{2,u}-\bar{z}_{2,(-)}}{\sigma_{2,(-)}}\right) - \Phi\left(\frac{\bar{z}_{2,l}-\bar{z}_{2,(-)}}{\sigma_{2,(-)}}\right)\right] & \text{if} \quad b^2 - 4ac \geq 0 \end{cases} \tag{S23}$$

where

$$C_{(+)} = \frac{1}{\sqrt{1+2\beta_1\sigma_2^2}} \exp\left\{\frac{-\beta_1\bar{z}_{2,F}^2 + (2\beta_1 z_\beta - \beta_3 \ln n_t)\bar{z}_{2,F} + (2\beta_1 z_\beta - \beta_3 \ln n_t)^2\sigma_2^2/2}{1+2\beta_1\sigma_2^2} + \beta_0 - \beta_1 z_\beta^2 - \beta_2 \ln n_t + \ln \Lambda_{S,t}\right\} \tag{S24}$$

$$C_{(-)} = \frac{1}{\sqrt{1-2\beta_1\sigma_2^2}} \exp\left\{\frac{\beta_1\bar{z}_{2,F}^2 - (2\beta_1 z_\beta - \beta_3 \ln n_t)\bar{z}_{2,F} + (2\beta_1 z_\beta - \beta_3 \ln n_t)^2\sigma_2^2/2}{1-2\beta_1\sigma_2^2} - \beta_0 + \beta_1 z_\beta^2 + \beta_2 \ln n_t - \ln \Lambda_{S,t}\right\} \tag{S25}$$

$$\bar{z}_{2,(+)} = \frac{\bar{z}_{2,F} + \sigma_2^2(2\beta_1 z_\beta - \beta_3 \ln n_t)}{1 + 2\beta_1\sigma_2^2} \tag{S26}$$

$$\bar{z}_{2,(-)} = \frac{\bar{z}_{2,F} - \sigma_2^2(2\beta_1 z_\beta - \beta_3 \ln n_t)}{1 - 2\beta_1\sigma_2^2} \tag{S27}$$

$$\sigma_{2,(+)} = \sqrt{\frac{\sigma_2^2}{1 + 2\beta_1\sigma_2^2}} \tag{S28}$$

$$\sigma_{2,(-)} = \sqrt{\frac{\sigma_2^2}{1 - 2\beta_1\sigma_2^2}} \tag{S29}$$

$$\bar{z}_{2,l} = z_\beta - \frac{\beta_3}{2\beta_1} - \frac{1}{2\beta_1}\sqrt{4\beta_1(\beta_0 - \beta_2 \ln n_t - \beta_3 z_\beta \ln n_t + \ln \Lambda_{S,t}) + \beta_3^2(\ln n_t)^2} \tag{S30}$$

$$\bar{z}_{2,u} = z_\beta - \frac{\beta_3}{2\beta_1} + \frac{1}{2\beta_1}\sqrt{4\beta_1(\beta_0 - \beta_2 \ln n_t - \beta_3 z_\beta \ln n_t + \ln \Lambda_{S,t}) + \beta_3^2(\ln n_t)^2}. \tag{S31}$$

The selection differential is

$$\Delta\bar{\mathbf{z}}_{S,t} = \begin{cases} \left[0, -\frac{\sigma_2^2}{1+2\beta_1\sigma_2^2}(2\beta_1(\bar{z}_{2,F} - z_\beta) + \beta_3 \ln n_t)\right]^T & \text{if} \quad b^2 - 4ac < 0 \\ \left[0, \left(\frac{1}{2}C_{(+)}\left\{\bar{z}_{2,(+)}\left[1 - \Phi\left(\frac{\bar{z}_{2,u}-\bar{z}_{2,(+)}}{\sigma_{2,(+)}}\right) + \Phi\left(\frac{\bar{z}_{2,l}-\bar{z}_{2,(+)}}{\sigma_{2,(+)}}\right)\right]\right.\right.\right. & \\ \quad \left. -\sigma_{2,(+)}\left[-\phi\left(\frac{\bar{z}_{2,u}-\bar{z}_{2,(+)}}{\sigma_{2,(+)}}\right) + \phi\left(\frac{\bar{z}_{2,l}-\bar{z}_{2,(+)}}{\sigma_{2,(+)}}\right)\right]\right\} & \\ \quad -\bar{z}_{2,F}\left[\Phi\left(\frac{\bar{z}_{2,u}-\bar{z}_{2,F}}{\sigma_2}\right) - \Phi\left(\frac{\bar{z}_{2,l}-\bar{z}_{2,F}}{\sigma_2}\right)\right] & \\ \quad -\sigma_2\left[\phi\left(\frac{\bar{z}_{2,u}-\bar{z}_{2,F}}{\sigma_2}\right) - \phi\left(\frac{\bar{z}_{2,l}-\bar{z}_{2,F}}{\sigma_2}\right)\right] & \\ \quad -\frac{1}{2}C_{(-)}\left\{\bar{z}_{2,(-)}\left[\Phi\left(\frac{\bar{z}_{2,u}-\bar{z}_{2,(-)}}{\sigma_{2,(-)}}\right) + \Phi\left(\frac{\bar{z}_{2,l}-\bar{z}_{2,(-)}}{\sigma_{2,(-)}}\right)\right]\right. & \\ \quad \left.\left. -\sigma_{2,(-)}\left[\phi\left(\frac{\bar{z}_{2,u}-\bar{z}_{2,(-)}}{\sigma_{2,(-)}}\right) + \phi\left(\frac{\bar{z}_{2,l}-\bar{z}_{2,(-)}}{\sigma_{2,(-)}}\right)\right]\right\}\right) & \\ \quad \left. /\bar{S}(\bar{\mathbf{z}}_{F,t}, n_t) - \bar{\mathbf{z}}_{F,t}\right]^T & \text{if} \quad b^2 - 4ac \geq 0 \end{cases} \tag{S32}$$

S2: Details regarding Figure 3

Each grid cell in both subfigures are constructed similarly:

1. Simulate 100 times the evolution of mean phenotypes $\bar{z}_1$ and $\bar{z}_2$, using the algorithm in the previous section, for 1000 generations ($t_{\max}$).
2. Compute the mean value of the mean phenotypes at the 1000th generation over the 100 iterations, i.e. $\bar{\mathbf{z}}_{1000} = \frac{1}{100}\sum_{j=1}^{100} \bar{\mathbf{z}}_{1000,(j)}$
3. Compute the difference between $\bar{\mathbf{z}}_{1000}$ for the current grid cell and the grid cell where $\sigma^2_{\text{fec}} = \sigma^2_{\text{sur}} = 0$ (bottom left corner of the figures) and divide it by the difference between the grid cell where $\sigma^2_{\text{fec}} = \sigma^2_{\text{sur}} = 1$ (top right corner) and where $\sigma^2_{\text{fec}} = \sigma^2_{\text{sur}} = 0$ (bottom left). This will give us differences in either $\bar{z}_1$ or $\bar{z}_2$ with respect to increases in σ^2_{fec} or σ^2_{sur} that are relative to largest difference, which occurs at the maximum value of the enivronmental noise. We denote this difference relative to the largest difference by $\tilde{\Delta}$.
4. In addition, we create a new variable which is the smallest value of the differenes in $\bar{z}_1$ or $\bar{z}_2$, thus for each combination of σ^2_{fec} and σ^2_{sur}, we have three values $[\tilde{\Delta}\bar{z}_1, \tilde{\Delta}\bar{z}_2, \min(\tilde{\Delta}\bar{z}_1, \tilde{\Delta}\bar{z}_2)]$.
5. Color each grid cell by applying:
   - a gradient of red colour with respect to $\tilde{\Delta}\bar{z}_1$, so that the colour is transparent (white) if $\tilde{\Delta}\bar{z}_1 = 0$ or full (red) if $\tilde{\Delta}\bar{z}_1 = 1$.
   - a gradient of blue colour with respect to $\tilde{\Delta}\bar{z}_2$, so that the colour is transparent (white) if $\tilde{\Delta}\bar{z}_2 = 0$ or full (blue) if $\tilde{\Delta}\bar{z}_2 = 1$.
   - a gradient of purple colour with respect to $\min(\tilde{\Delta}\bar{z}_1, \tilde{\Delta}\bar{z}_2)$, so that the colour is transparent (white) if $\min(\tilde{\Delta}\bar{z}_1, \tilde{\Delta}\bar{z}_2) = 0$ or full (purple) if $\min(\tilde{\Delta}\bar{z}_1, \tilde{\Delta}\bar{z}_2) = 1$.

This computation and coloring scheme will result in a grid where cells are white if there is no change in either $\bar{z}_1$ or $\bar{z}_2$ compared to a population with no environmental variance ($\tilde{\Delta}\bar{z}_1 = \tilde{\Delta}\bar{z}_2 = \min(\tilde{\Delta}\bar{z}_1, \tilde{\Delta}\bar{z}_2) \approx 1$). The cell will have a red color if the change is mostly in the $\bar{z}_1$ direction ($\tilde{\Delta}\bar{z}_1 \approx 1$ and $\tilde{\Delta}\bar{z}_2 \approx 0$), a blue color if the change is mostly in the $\bar{z}_2$ ($\tilde{\Delta}\bar{z}_1 \approx 0$ and $\tilde{\Delta}\bar{z}_2 \approx 1$) direction and a more purple color if both $\bar{z}_1$ and $\bar{z}_2$ are changing rather similarly ($\min(\tilde{\Delta}\bar{z}_1, \tilde{\Delta}\bar{z}_2) \approx 1$).

In both subfigures $\sigma_1 = 1$, $\sigma_2 = 1$, $\rho = 0$, $G_{11} = 1$, $G_{12} = G_{21} = 0$ and $G_{22} = 1$. Both subfigures also have $\alpha_0 = 7$, $\alpha_1 = 0.001$, $\alpha_3 = 0.005$, $\beta_0 = 5$, $\beta_1 = 0.001$ and $\beta_3 = 0.005$. The environmental variance in fecundity (σ^2_{fec}) and survival (σ^2_{sur}) are increasing from 0 to 1 by increments of 0.1. **A** we have $\alpha_2 = 0.5$ and $\beta_2 = 0.5$. **B** we have $\alpha_2 = 1$ and $\beta_2 = 1$.
